# An Embedding 2D/3D Heterostructure Enables High‐Performance FA‐Alloyed Flexible Perovskite Solar Cells with Efficiency over 20%

**DOI:** 10.1002/advs.202101856

**Published:** 2021-10-08

**Authors:** Zhen Wang, Yuanlin Lu, Zhenhua Xu, Jinlong Hu, Yijun Chen, Cuiling Zhang, Yousheng Wang, Fei Guo, Yaohua Mai

**Affiliations:** ^1^ Institute of New Energy Technology College of Information Science and Technology Jinan University Guangzhou 510632 China; ^2^ Institute of Polymer Optoelectronic Materials & Devices State Key Laboratory of Luminescent Materials & Devices South China University of Technology Guangzhou 510640 China; ^3^ Key Laboratory of Advanced Material Processing and Mold (Ministry of Education) Zhengzhou University Zhengzhou 450002 China

**Keywords:** 2D/3D heterostructures, flexible perovskite solar cells, mechanical stability, nonradiative recombination

## Abstract

Flexible perovskite solar cells (*f*‐PSCs) have attracted increasing attention because of their enormous potential for use in consumer electronic devices. The key to achieve high device performance is to deposit pinhole‐free, uniform and defect‐less perovskite films on the rough surface of polymeric substrates. Here, a solvent engineering to tailor the crystal morphology of FA‐alloyed perovskite films prepared by one‐step blade coating is first deployed. It is found that the use of binary solvents DMF:NMP, rather than the conventional DMF:DMSO, enables to deposit dense and uniform FA‐alloyed perovskite films on both the rigid and flexible substrates. As a decisive step, an embedding 2D/3D perovskite heterostructure is in situ formed by incorporating a small amount of 4‐guanidinobutanoic acid (GBA). Accordingly, photovoltage increases up to 100 mV are realized due to the markedly suppressed nonradiative recombination, leading to high efficiencies of 21.45% and 20.16% on the rigid and flexible substrates, respectively. In parallel, improved mechanical robustness of the flexible devices is achieved due to the presence of the embedded 2D phases. The results underpin the importance of morphology control and defect passivation in delivering high‐performance flexible FA‐alloyed flexible perovskite devices.

## Graphical abstract

An embedding 2D/3D heterostructure is in situ formed by incorporating a small amount of 4‐guanidinobutanoic acid, which markedly suppresses nonradiative recombination, leading to high efficiencies of 21.45% and 20.16% for the rigid and flexible perovskite devices, respectively.

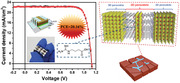

## 1 Introduction

In recent years, organic–inorganic hybrid perovskites have drawn tremendous attention for photovoltaic applications due to their intriguing properties, such as high carrier mobility, high absorption coefficients, long diffusion lengths, and low exciton binding energy.^[^ ^1^, ^2^, ^3^, ^4^ ^]^ The highest power conversion efficiency (PCE) of perovskite solar cells (PSCs) has reached a high level of 25.5%.^[^ ^5^ ^]^ A noteworthy advantage of PSCs is that they can be solution processed at low temperatures, which is of particular interest to fabricate flexible devices.^[^ ^6^ ^]^ Indeed, recent years have witnessed the rapid advance of *f*‐PSCs with the highest PCE exceeding 20%, illustrating great commercial potentials for use in wearable electronics, intelligent vehicles, and building‐integrated photovoltaics, etc.^[^ ^7^, ^8^, ^9^, ^10^ ^]^

Nevertheless, it should be noted that the efficiency of *f*‐PSCs still lags far behind those of rigid devices. The major challenge originated from the difficulty to deposit uniform and pinhole‐free perovskite on flexible substrates, primarily due to the fact that crystallization dynamics on the high rough surface of polymeric substrates is more difficult to be controlled than on the rigid glass. To surmount these deficiencies, engineering perovskite precursor inks with improved rheological properties by incorporating processing additives, such as polymers, surfactants and mixed solvents, have been proposed to deposit dense and pinhole‐free perovskite films on flexible substrates.^[^ ^11^, ^12^, ^13^, ^14^, ^15^, ^16^, ^17^ ^]^ On the other hand, large quantity of electronic defects located at the surface and grain boundaries of the perovskite crystals can serve as nonradiative charge‐recombination centers, thereby limiting the achievable photovoltage of the devices. Recent works have demonstrated molecular passivation strategies to heal the defects at surface and grain boundaries of perovskites, leading to improved efficiency and stability of *f*‐PSCs.^[^ ^18^, ^19^, ^20^, ^21^, ^22^ ^]^ Nevertheless, the majority of these high efficiency *f*‐PSCs were deposited using spin coating in combination with an antisolvent crystallization process, which is unfortunately neither scalable nor can be transferred to large‐area coating lines. In this circumstance, developing effective strategies allowing for morphology control and defect passivation of flexible perovskite films by scalable deposition process is highly demanded for their large‐scale manufacture.

In this paper, we report a scalable approach to fabricate high‐performance FA‐alloyed *f*‐PSCs by a synergetic morphology and defects control via solvent and dimensionality engineering, respectively. As a first step, dense and pinhole‐free FA‐alloyed perovskites with large grains are readily prepared by using a binary solvent DMF:NMP. Inverted rigid PSCs processed from the DMF:NMP yield a decent PCE of 18.49%. We further introduce a small amount of 4‐guanidinobutanoic acid (GBA) into the perovskite precursor, thereby in situ forming an embedded 2D/3D heterostructure. The vertically aligned 2D perovskites effectively passivate the electronic defects at grain boundaries of 3D crystals. As a result, the open‐circuit voltage (*V*_OC_) of the 2D/3D heterostructure PSCs increases by ≈100 mV as compared to the control devices, yielding PCEs over 21% for the rigid devices. In parallel, *f*‐PSCs based on the embedding 2D/3D structure yield high efficiencies of 20.16% and 16.86% for the device areas of 0.09 and 1 cm^2^, respectively. Moreover, the mechanical stability of the *f*‐PSCs based on the 2D/3D heterostructure is also noticeably enhanced.

## 2 Results and Discussion

### 2.1 Morphology Optimization of FA‐Alloyed Perovskites via Solvent Engineering

We choose a perovskite material, FA_0.7_MA_0.25_Cs_0.05_Pb(I_0.93_Br_0.07_)_3_, with mixed cations of methylammonium (MA), formamidinium (FA), and caesium (Cs) as the absorbers of our photovoltaic devices owing to their relatively smaller bandgaps (*E*_g_), better stability, and higher efficiencies compared to the MAPbI_3_.^[^ ^16^, ^23^, ^24^ ^]^ All the perovskite films were deposited using a vacuum‐assisted blade coating, which was recently developed in our lab as a scalable manufacturing process for high qualitative perovskite layers.^[^ ^15^, ^25^, ^26^ ^]^ As illustrated in Figure S1, Supporting Information, a perovskite precursor wet film is first blade coated on the rigid or flexible substrate at room temperature. Subsequently, the obtained precursor film is subjected to a mild vacuum of 1000 Pa for 90 s, with which an intermediate phase film is obtained. Finally, crystalline perovskite films are obtained by annealing the resulting intermediate film at 100 °C for 15 min.

**Figure 1:**
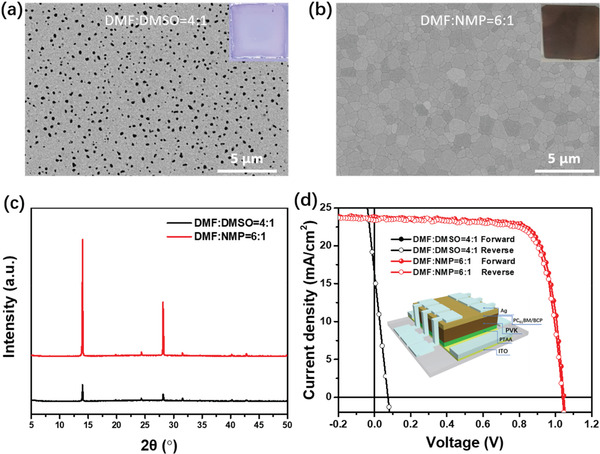
SEM images of the FA‐alloyed perovskite films based on the two solvent systems: a) DMF:DMSO = 4:1 and b) DMF:NMP = 6:1 (volume ratio); c) XRD patterns of the corresponding perovskite films processed with two solvent; d) *J–V* curves of PSCs based on mixed solvent for DMF:DMSO = 4:1 and DMF:NMP = 6:1, and the inset is schematic diagram of photovoltaic device architecture.

It is well recognized that the morphology, crystallinity, and grain size of the perovskite films are closely correlated with photovoltaic performance of PSCs. The Lewis acid–base adduct approach is one of the effective methods to regulate the nucleation and crystal growth, thereby tailoring the morphology and electronic quality of perovskite films.^[^ ^27^, ^28^, ^29^ ^]^ For example, the combination of dimethyl sulfoxide (DMSO) and DMF has been established as a standard solvent system to produce high‐quality MA‐only and mixed‐cation perovskite films by spin coating.^[^ ^30^, ^31^, ^32^, ^33^ ^]^ However, we find that, using the conventional binary solvent of DMF:DMSO, it is unable to produce qualified FA‐containing mixed‐cation perovskite layers neither on rigid nor flexible substrates by our vacuum‐assisted blade coating. As shown in **Figure** **1**a, small grains with massive pinholes are observed in the perovskite films processed from the DMF:DMSO with volume ratio of 4:1.

We therefore re‐design the precursor ink by replacing the commonly used DMSO with NMP to regulate the crystallization of the FA‐alloyed perovskite films prepared by vacuum‐assisted blade coating.^[^ ^15^, ^25^, ^34^ ^]^ The choice of NMP as a co‐solvent is mainly based on the fact that NMP has a stronger interaction with the FA cation than the DMSO.^[^ ^35^, ^36^ ^]^ In addition, NMP has a lower vapor pressure (0.29 mmHg at 20 °C) than that of the DMSO (0.42 mmHg), which facilitates the formation of a stable FAI·PbI_2_·NMP adduct. As a result, the crystallization rate can be slowed down by using the solvent of DMF:NMP, resulting in dense and uniform FA‐alloyed perovskite films. As shown in Figure 1b and Figure S2, Supporting Information, compact and pinhole‐free perovskite films with large grains are readily obtained by using DMF:NMP binary solvent of various volume ratios. Figure 1c shows the X‐ray diffraction(XRD) spectra of the corresponding blade‐coated FA‐alloyed perovskite films with different solvent systems. Compared to the film deposited from DMF:DMSO, the perovskite film processed from DMF:NMP binary solvent exhibits a much stronger (110) diffraction peak along with a narrower half‐peak width, attesting significantly enhanced crystallinity of the DMF:NMP‐based perovskite film.

Photovoltaic performance of the FA‐alloyed perovskites processed from the two solvent systems was evaluated by constructing solar devices with a layer stack of “ITO/PTAA/Perovskite/PCBM/BCP/Ag.” The corresponding *J–V* characteristics are shown in Figure 1d and Figure S3, Supporting Information with the detailed device parameters summarized in Tables S1 and S2, Supporting Information. Not surprisingly, the device processed from DMF:DMSO is almost shorted due to the presence of a number of pinholes, giving a rather low efficiency of 0.3%. In distinct contrast, all the devices processed from the DMF:NMP solvent show excellent diode characteristics. It is seen that the *V*_OC_, short‐circuit current density (*J*_SC_), and FF of the device is significantly increased when NMP is added to the DMF with ratio of 1:8, resulting in a high PCE of 17.77%. With increase of NMP to 6:1, a higher *J*_SC_ over 23 mA cm^−2^ is obtained, delivering a champion PCE of 18.49%. However, further increase the content of NMP to 4:1, *J*_SC_ decreases to ≈21 mA cm^−2^, declining the PCE to ≈16%. The corresponding statistic photovoltaic parameters are shown in the Figure S4, Supporting Information, indicating good reproducibility of the devices. The external quantum efficiency (EQE) spectrum of the champion PSC processed from DMF:NMP = 6:1 is shown in Figure S5, Supporting Information. The integration of the EQE with AM 1.5 G gives an *J*_SC_ value of 23.19 mA cm^−2^, which is in good agreement with the *J–V* measurement, with a small discrepancy below 5%. Overall, these results demonstrate that the incorporation of NMP plays a determinative role in obtaining dense and uniform FA‐alloyed perovskite films, which is of highly relevance for achieving high device performance.

### 2.2 In Situ Forming an Embedding 2D/3D Heterostructure

**Figure 2:**
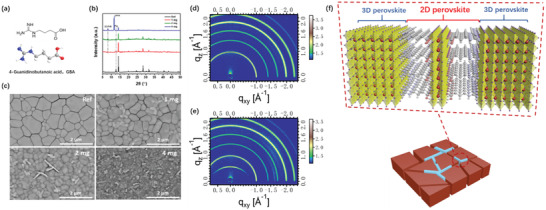
a) Molecular formula (top) and ball‐and‐stick model (bottom) of GBA; b) XRD spectra for perovskite films prepared by precursor solution with different concentration of GBA (mg mL^−1^); c) SEM images of perovskite films prepared via precursor solution with different concentration of GBA on the glass substrates; The GIWAXS images of FA_0.7_MA_0.25_Cs_0.05_Pb(I_0.93_Br_0.07_)_3_ for d) reference and e) containing 2 mg mL^−1^ GBA; f) Schematic illustration of 2D/3D perovskite model.

Although the photovoltaic performance of the FA‐alloyed PSCs has been markedly improved by using the binary solvent DMF:NMP, it is noticed that there is a large photovoltage deficit (*E*_g −_ *V*_OC_ = 0.52 eV), which is a major contributor limiting the device efficiency. The large photovoltage losses can be primarily ascribed to the severe nonradiative recombination due to the presence of deep‐level trap states. Previous studies have illustrated that constructing 2D/3D heterostructure is an effective means to suppress defect‐assisted nonradiative recombination in perovskite devices.^[^ ^26^, ^37^ ^]^ To this purpose, we incorporate a small amount of guanidine derivative, GBA (**Figure** **2**a), into the FA_0.7_MA_0.25_Cs_0.05_Pb(I_0.93_Br_0.07_)_3_ precursor solution to prepare 2D/3D perovskite films. The selection of GBA as bulky organic cations of 2D perovskites is mainly based on the fact that the existence of three nitrogen atoms in GBA enables to passivate the deep‐level antisite defects by forming strong bonding between the electron lone pairs of nitrogen and the undercoordinated metal cations.^[^ ^38^ ^]^

Figure 2b shows the XRD spectra of the FA‐alloyed perovskite films with and without addition of GBA. It is seen that undoped perovskite film possesses a standard *α*‐phase with no hexagonal yellow phase (*δ*‐phase) being detected. A small diffraction peak at 12.7° is assigned to the excessive PbI_2_ that are intentionally added into the precursor. The control perovskite film with excessive PbI_2_ shows a similar crystal morphology as compared to the film processed from the stoichiometric precursor (Figure 2b). With addition of a small amount of GBA (1 mg mL^−1^), a new weak peak at 2*θ* = 8.04° is observed, which can be ascribed to the formation of 2D perovskite phase.^[^ ^37^ ^]^ The corresponding SEM image evidences some new phases with irregular shapes embed among grain boundaries (Figure 2c). Meanwhile, the grain size become smaller with incorporation of GBA, which can be ascribed to the fact that the growth of 3D crystals can be inhibited by the GBA molecules. Further increasing content of GBA to 2 mg mL^−1^, the characteristic peak (2*θ* = 8.04°) of 2D perovskite becomes stronger, suggesting the growth of more 2D phases. The corresponding SEM image shown in Figure 2c evidences more smaller 3D crystals with several 2D sheets vertically embedded in the 3D grains. We note that similar vertical alignment of 2D perovskite sheets in 3D crystals have also been observed in earlier reports.^[^ ^37^, ^39^ ^]^ However, when the concentration of GBA increases to 4 mg mL^−1^, an evident and intensive diffraction peak at 11.7° appears, which can be due to the formation of *δ*‐phase. The resulting perovskite film contains mainly fragmentized and unordered crystalline grains. These observations imply that excessive GBA can destroy the crystal structure of 3D perovskite and transform it to layered *δ*‐phase structure.

To confirm the crystal structure and orientation of the perovskite films, we recorded the grazing‐incidence wide‐angle X‐ray scattering (GIWAX) patterns of the FA_0.7_MA_0.25_Cs_0.05_Pb(I_0.93_Br_0.07_)_3_ processed with and without addition of 2 mg mL^−1^ GBA. As presented in Figure 2d, several major diffraction rings at *q*_z_>1 A^−1^ are observed in the two perovskite films, indicating that the perovskite crystals are oriented in random directions.^[^ ^26^ ^]^ Noticeably, a new diffraction ring at *q*_z_ = 0.57 Å^−1^ in out‐of‐plane direction is observed when 2 mg mL^−1^ GBA is incorporated (Figure 2e and Figure S6, Supporting Information), confirming the formation of 2D layered perovskite with a *d*‐spacing of 11.02 Å. Overall, with all the above structure and morphology characterizations of the perovskite films with addition of GBA, we schematically depict the model of the resulting 2D/3D perovskite heterostructure which is displayed in Figure 2f: the layered 2D perovskite sheets are mainly vertically embedded at the grain boundaries of the bulk 3D perovskite crystals.

**Figure 3:**
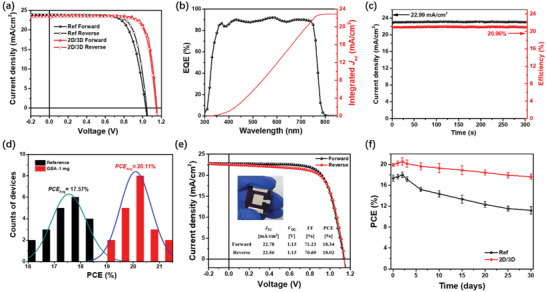
a) *J‒V* curves of the best‐performing PSCs without and with 1 mg mL^−1^ GBA. b) EQE spectra of the best‐performing 2D/3D perovskite solar cells. c) The steady‐state PCE and *J*_sc_ of the champion 2D/3D perovskite solar cells. d) PCE histograms of the reference and the 2D/3D PSC. e) *J‒V* curves of the large area (1 cm^2^) 2D/3D perovskite solar cells. Inset shows the photo of the device. f) Shelf stability of the unencapsulated PSCs stored in N_2_ glovebox.

To examine the effectiveness of 2D perovskite passivation on the photovoltaic properties, we fabricated perovskite devices on rigid ITO‐coated glass substrates. The photovoltaic parameters of the devices processed from different concentrations of GBA are listed in **Table** **1** with the corresponding statistic parameters exhibited in Figure S8, Supporting Information. It is apparent that the efficiency of the devices is distinctly improved from 18.51% for the control device to 20.22% with addition of only 0.5 mg mL^−1^ GBA, which mainly arises from the *V*_OC_ enhancement (from 1.04 to 1.11 V). With addition of 1 mg mL^−1^ GBA, the *V*_OC_ of the 2D/3D perovskite devices is further increased to 1.14 V along with a high FF of 80%, yielding a champion PCE of 21.45% (**Figure** **3**a). Besides, hysteresis is suppressed in the 2D/3D PSCs as compared to the reference device, which is probably due to the fact that the 2D perovskite embedded at grain boundaries of 3D grains can block ion diffusion in perovskite lattice.^[^ ^40^ ^]^ Further increasing the concentration of GBA to 2 mg mL^−1^, both the *V*_OC_ and FF start to decline, resulting in a low PCE of 19.11%. However, *J*_SC_ drops dramatically to less than 9 mA cm^−2^ when the concentration of GBA increases to 4 mg mL^−1^, which can be attributed to the emergence of *δ*‐phase perovskites that seriously impairs charge generation and transport.

**Table 1:** Photovoltaic performance parameters of the PSCs fabricated from precursor solutions containing different concentrations of GBA

GBA concentration	Scan	*J*_SC_	*V*_OC_	FF	PCE_Max_	PCE_Avg_
[mg mL^−1^]	direction	[mA cm^−2^]	[V]	[%]	[%]	[%]
0 (Ref)	Forward	23.84	1.03	71.12	17.46	17.57
	Reverse	23.89	1.04	74.50	18.51	
0.5	Forward	23.62	1.11	77.13	20.22	19.65
	Reverse	23.57	1.11	77.20	20.19	
1	Forward	23.23	1.14	79.66	21.10	20.11
	Reverse	23.51	1.14	80.02	21.45	
2	Forward	23.00	1.10	75.54	19.11	18.84
	Reverse	22.95	1.10	75.13	18.97	
4	Forward	8.85	1.03	60.47	5.51	4.25
	Reverse	8.68	1.03	58.11	5.20	

The integration of the EQE spectra gives a current density of 22.87 mA cm^−2^ for the PSCs fabricated from precursor solution of 1 mg mL^−1^ GBA (Figure 3b), confirm the reliability of the *J–V* measurement. Figure 3c shows the steady‐state output photocurrent and efficiency of the best‐performing PSC recorded at the maximum power point of 0.98 V for 300 s. The PCE of the device stabilizes at 20.96%, which is close to the value obtained from the *J‒V* measurement. The PCE histogram combined with the Gaussian fitting of the reference and 2D/3D PSCs is illustrated in Figure 3d. The average PCE values of PSCs are significantly enhanced from 17.57% (reference) to 20.11% (2D/3D). In addition, all of the 2D/3D PSCs yield PCE values noticeably higher than the maximum PCE of the control devices. To demonstrate the scalability of the 2D/3D heterojunction strategy, PSCs with an active area of 1 cm^2^ were also fabricated. The best device yields a high PCE of 18.34% with an FF of 71.23%. These results validate the effectiveness and excellent reproducibility of the 2D/3D perovskites heterostructure in delivering high device performance.

We also evaluated the shelf stability of our PSCs which were stored in the N_2_‐filled glovebox without encapsulation. As shown in the Figure 3f, there is a slight but distinct PCE increase at the initial storage for both the reference and the 2D/3D PSCs, which is likely due to improved contact within the device stack.^[^ ^41^, ^42^ ^]^ Afterward, efficiencies begin to decline, which maintain 89% and 64% after 30 days’ storage for the 2D/3D heterostructure cell and reference device, respectively.

**Figure 4:**
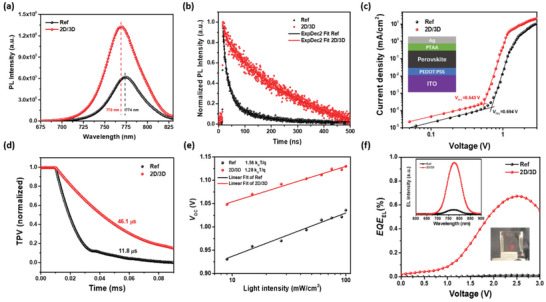
a) Steady‐state PL of the reference and 2D/3D perovskite films; b) TRPL decay curves for the reference and 2D/3D perovskite films; c) Logarithm of *J–V* curves in the dark for the reference and the 2D/3D devices (inset shows the device structure); d) Transient photovoltage decay curves of the reference and 2D/3D PSCs; e) *V*_OC_ as a function of illumination light intensity for the reference and the 2D/3D PSCs; f) EQE electroluminescence as a function of voltage for the reference and the 2D/3D devices, the left inset is the corresponding EL spectra; the right inset shows the 2D/3D PSC operating as an LED, which emits clearly visible red light.

To elucidate the origin of the markedly enhanced performance of the 2D/3D heterostructure PSCs, we first evaluated the charge dynamics in the perovskite films. **Figure** **4**a shows the steady‐state photoluminescence (PL) of the reference and the 2D/3D perovskite films. The PL emission intensity of the 2D/3D heterostructure film is ≈2 times stronger than the reference, indicating significantly suppressed nonradiative recombination in 2D/3D film.^[^ ^43^ ^]^ Moreover, a slight blue shift (from 774 to 770 nm) of the emission peak for 2D/3D heterostructure perovskite film is observed, which can be ascribed to the alleviated energetic disorder as a result of removing tail states by defect passivation.^[^ ^44^ ^]^ The time‐resolved PL (TRPL) spectra in Figure 4b shows that the 2D/3D perovskite film exhibits a significantly longer carrier lifetime (367.27 ns) than the reference film (28.21 nm) (Table S3, Supporting Information), indicating reduced defect state density in 2D/3D film. To quantitatively determine the trap‐state density of the perovskite films, hole‐only devices with a device configuration of ITO/PEDOT:PSS/perovskite/PTAA/Ag were fabricated (Figure 4c). The trap‐state densities are calculated according to the below formula.^[^ ^45^ ^]^

(1) $$N_{\mathrm{trap}}=\;\frac{2\varepsilon \varepsilon_{0}V_{\mathrm{TFL}}}{qL^{2}}$$

where *ɛ* is the relative dielectric constant of perovskite, and *ɛ*_0_ is the permittivity of vacuum, *q* is the elemental charge. *V*_TFL_ is the trap‐filling limited voltage, and *L* is the thickness of films (according to Figure S9, Supporting Information, *d* ≈300 nm). The trap‐state density is calculated to be 2.34 × 10^−16^ cm^−1^ for the 2D/3D heterostructure perovskite, which is much higher than that of the reference film (2.98 × 10^−16^ cm^−1^). These results suggest that the trap density in the 2D/3D heterostructure perovskite film is significantly decreased, which is particularly beneficial for suppressing nonradiative recombination in solar cells.

To gain deeper insight into the passivation mechanism of GBA on the perovskite films, X‐ray photoelectron spectroscopy (XPS) was performed. It is seen that two intensive peaks at 138.3 and 143.2 eV indexing to the Pb 4f_7/2_ and Pb 4f_5/2_ are observed in the 3D reference film, which are shifted to 137.8 and 142.7 eV in the 2D/3D perovskite films, respectively (Figure S10a, Supporting Information). This can be ascribed to the donation of the lone electron pair on the nitrogen (N) atom to the Pb^2+^ via coordination bonding.^[^ ^26^ ^]^ Similarly, the peak assigned to O 1s is shifted from 531.0 eV (pure GBA) to 532.1 eV (2D/3D perovskite) (Figure S10b, Supporting Information). Meanwhile, the N 1s peak of the 2D/3D film shifts toward higher binding energy compared to pure GBA (Figure S10c, Supporting Information). These observations suggest strong coordinating interaction between the GBA molecules and Pb^2+^, allowing to suppress Pb vacancy and Pb–I antisite defects in the 2D/3D heterostructure film.^[^ ^46^, ^47^ ^]^

We also evaluated the charge‐recombination dynamics in the solar devices by performing transient photovoltage (TPV) measurements. It is seen that the 2D/3D perovskite device exhibits a longer charge‐recombination lifetime (46.1 µs) as compared to the reference device (11.8 µs) (Figure 4d). The longer carrier decay time indicates slower charge recombination, which can be the main factor responsible for the enhanced *V*_OC_ of 2D/3D PSCs. Figure 4e plots the *V*_OC_ dependence on the light intensity of the two devices. The slope of *V*_OC_ versus the natural logarithm of light intensity for 2D/3D device is determined to be 1.28 *k*_B_ *T*/*q*, which is lower than that of the reference device (1.56 *k*_B_ *T*/*q*). This result demonstrates that trap‐assisted charge recombination is effectively suppressed in the 2D/3D perovskite films.

The above combinational characterizations confirm that nonradiative recombination is markedly suppressed in the 2D/3D heterostructure perovskite films, which contributes to the significantly enhanced *V*_OC_ (from 1.04 to 1.14 V) of the devices. In order to quantitatively evaluate the *V*_OC_ losses, we operated the two PSCs as LEDs to examine their electroluminescence (EL) performance (Figure 4f). An EL spectrum at 776 nm is observed for both the reference and 2D/3D devices, and the intensity of EL emission of the 2D/3D device is significantly stronger than the reference device, indicating that nonradiative recombination channels in the 2D/3D perovskite is greatly suppressed. According to the Shockley–Queisser detailed balance theory, the *V*_OC_ loss in solar cells can be divided into radiative loss and nonradiative loss, as shown in Equation (2).^[^ ^48^ ^]^

(2) $$\begin{array}{ccc} \Delta V_{\mathrm{OC}} & = & \frac{E_{g}}{q}-V_{\mathrm{OC}}=\left(\frac{E_{g}}{q}-V_{\mathrm{OC}}^{\mathrm{rad}}\right)+\left(V_{\mathrm{OC}}^{\mathrm{rad}}-V_{OC}\right)\\ & = & \Delta V_{\mathrm{OC}}^{\mathrm{rad}}+\Delta V_{\mathrm{OC}}^{\mathrm{nonrad}} \end{array}$$

(3) $$\Delta V_{\mathrm{OC}}^{\mathrm{nonrad}}=\;-\frac{kT}{q}In\;EQE_{\mathrm{EL}}$$

where *E*_g_ is the bandgap energy; *q* is the elemental charge; *k* is the Boltzmann constant; and *T* is the Kelvin temperature. $\Delta V_{\mathrm{OC}}^{\mathrm{rad}}$ and $\Delta V_{\mathrm{OC}}^{\mathrm{nonrad}}$ are the *V*_OC_ losses via radiative recombination and nonradiative recombination, respectively. The first term due to the radiative recombination is unavoidable,^[^ ^49^ ^]^ thereby $\Delta V_{\mathrm{OC}}^{\mathrm{nonrad}}$ should be minimized to achieve high *V*_OC_ values. For reference device, the *EQE*_EL_ of 0.012% is attained at a driving current density of 22.11 mA cm^−2^ that is equivalent to the *J*_SC_ (Figure S11, Supporting Information). The calculated nonradiative *V*_OC_ loss is 233 mV.^[^ ^49^ ^]^ In contrast, the 2D/3D PSC shows a higher *EQE*_EL_ of 0.42% at the current density of 23.54 mA cm^−2^, giving to a much smaller nonradiative *V*_OC_ loss of 142 mV.

### 2.3 High‐Efficiency Flexible PSCs Based on the 2D/3D Heterostructure

**Figure 5:**
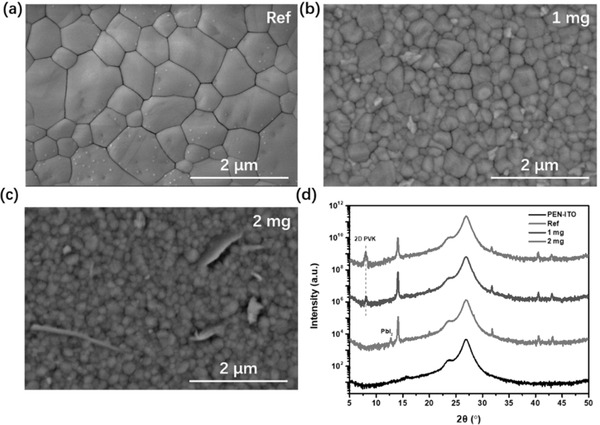
a–c) SEM images and d) XRD spectra of perovskite films blade coated on the flexible PEN/ITO substrates, which are processed from precursor solutions of different amounts of GBA (mg mL^−1^).

To demonstrate the viability of the resulting 2D/3D heterostructure for the construction of high‐performance flexible PSCs, we first examined the crystal morphology and crystal structure of the FA‐alloyed perovskite films deposited on flexible PEN/ITO substrates. As shown in **Figure** **5**a, a compact perovskite film with large grains is readily obtained for reference sample, analogous to the films deposited on the rigid glass substrate. In particular, some new phases with irregular shapes are embed in the grain boundaries of 3D perovskite grains with addition of 1 mg mL^−1^ GBA (Figure 5b), indicating the formation of 2D/3D heterostructure on the polymeric substrate. Similar to the perovskite layers deposited on the rigid glass, a weak diffraction peak at 2*θ* = 8.04^o^ is observed, further confirming the formation of low‐dimensional 2D perovskite phase on the flexible substrate (Figure 5d). As the 2D phase increases by increasing the content of GBA (Figure 5d), the grains size of 3D perovskite further decreases (Figure 5c). This tendency coincidences with the perovskite deposited on rigid glass, suggesting that the developed 2D/3D heterostructure is readily applicable for the preparation of *f*‐PSCs and does not need to re‐formulate the precursor inks.^[^ ^50^ ^]^

**Figure 6:**
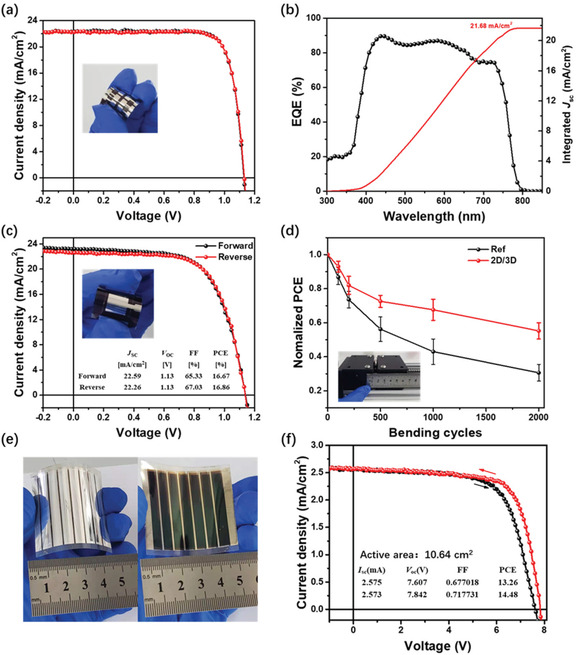
a) *J‒V* curves of the best‐performing 2D/3D *f*‐PSCs, inset shows the photo of flexible device; b) EQE spectra of best‐performing 2D/3D *f*‐PSCs; c) *J‒V* curves of a large‐area (1 cm^2^) 2D/3D *f*‐PSCs, inset shows the photo of the device; d) Normalized PCE evolution of the *f*‐PSCs as a function of bending cycles, the devices were bended at a small curvature radius of 3 mm; e) Photograph and f) *J−V* curve for flexible perovskite solar module.

We evaluated the photovoltaic performance of the *f*‐PSCs by constructing a layer stack of PEN/ITO/PTAA/perovskite/PC_61_BM/BCP/Ag. Figure S13 and Table S4, Supporting Information present the statistic photovoltaic parameters of the flexible devices without and with addition of different amounts of GBA. The flexible devices without GBA yield the highest PCE of 17.83% with an average value of 16.66%. With addition of GBA to 1 mg mL^−1^, a champion PCE of 20.16% is achieved with a high *V*_OC_ of 1.13 V (**Figure** **6**a). The corresponding EQE curve is presented in Figure 6b, giving an integrated *J*_SC_ of 21.68 mA cm^−2^. The slightly lower *J*_SC_ of the flexible device can be due to the fact that part of solar photons are absorbed by the flexible PEN/ITO substrates (Figure S14, Supporting Information).^[^ ^15^ ^]^ From Figure S15, Supporting Information, it is seen that absorption intensity of the perovskite film deposited on PEN substrate in the wavelength range from 300 to 350 nm is higher than that on the rigid glass, which mainly comes from the light absorption by the PEN substrate. It is also noticed that the absorption of the flexible perovskite film begins to dramatically decrease at the wavelength over 350 nm, which agrees well with EQE descending trend in wavelength over 400 nm in flexible device. Similar to the rigid PSCs, both the *J*_SC_ and *V*_OC_ start to decline when the concentration of GBA is increased to 2 mg mL^−1^, yielding an inferior PCE of 17.57% (Figure S12b, Supporting Information).

To further examine the scalability of the 2D/3D heterostructure on flexible substrates, *f*‐PSCs with an active area of 1 cm^2^ were fabricated, and the best device yields a high PCE of 16.86% with an FF of 67.03% (Figure 6c). We also evaluated the mechanical stabilities of our flexible PSCs by continuously bending the solar cells at a small curvature radius of 3 mm. As shown in Figure 6d, the *f*‐PSC based on the 2D/3D heterostructure retains over 55% of its original PCE after 2000 cycles. In contrast, the reference device maintains less than 30% of its initial PCE. These results demonstrate that the 2D/3D heterostructure perovskite is beneficial for improving the mechanical stability of *f*‐PSCs, which is likely due to the fact that the 2D sheets embedded in the 3D perovskite grains enable to release mechanical stress due to the presence of organic cations (Figure 2f).^[^ ^51^, ^52^ ^]^

Finally, the upscaling viability of the technology was further examined by manufacturing large‐area modules. Figures S17a and 6e, Supporting Information show the photographs of the prepared rigid and flexible solar module each comprising seven subcells connected in series, respectively. The area of each subcell is 0.38 × 4 cm^2^ including the interconnection area of 0.22 × 4 cm^2^, giving a total active area of 10.64 cm^2^. The rigid perovskite module gives a PCE of 16.03%, with a high *V*_OC_ of 7.94 V, a *J*_SC_ of 2.72 mA cm^−2^ and a high FF of 0.74 from the forward scan (Figure S17b, Supporting Information). The flexible solar module prepared on PEN substrate yields a champion PCE of 14.48%, along with a *V*_OC_ of 7.84V, a *J*_SC_ of 2.57 mA cm^−2^, and FF of 0.71 (Figure 6f). We note that this is one of the highest efficiencies achieved for flexible perovskite modules.^[^ ^53^, ^54^ ^]^

## 3 Conclusion

In summary, we have developed an effective solvent engineering for the deposition of dense and uniform FA‐alloyed perovskite films on both rigid and flexible substrates by scalable printing process. In combination with a rational dimensionality control by constructing an embedding 2D/3D heterostructure, the trap‐state defects of both the rigid and flexible perovskites can be effectively suppressed. As a result, nonradiative charge recombination is significantly reduced, which translates to an increase in *V*_OC_ by up to 100 mV. Ultimately, a high efficiency of 20.14% is achieved for the flexible perovskite devices, which is among the highest efficiencies reported in the literature. In parallel, the *f*‐PSCs based on the 2D/3D heterostructure exhibit better mechanical durability as compared to the reference 3D devices. Our work not only provides an effective method for controlling the morphology of scalable deposited FA‐alloyed perovskites but also underlines the importance of defects passivation in fabricating high‐performance flexible perovskite solar devices.

## 4 Experimental Section

### Materials

Lead iodide (PbI_2_, 99.9985%) were purchased from Alfa Aesar. Methylammonium iodide (MAI), poly[bis(4‐phenyl) (2,4,6‐trimethylphenyl)amine] (PTAA) were purchased from Xi'an p‐OLED Co (China). Thiourea (TU, >99.0%) were purchased from Tokyo Chemical Industry (TCI). Bathocuproine (BCP) and PC_61_BM were purchased from Lumtec. 4‐GBA were purchased from Bide Pharmatech. Ltd. All the solvents were purchased from Sigma‐Aldrich and all the chemicals were used as received without further purification. Flexible PEN/ITO and glass/ITO substrates were purchased from OPV Tech New Energy Co., Ltd.

### Blade Deposition of Perovskite Films

The FA_0.7_MA_0.25_Cs_0.05_Pb(I_0.93_Br_0.07_)_3_ precursor solution was prepared by dissolving 0.5 mol FAI, 0.18 mol MABr, 0.68 mol PbI_2_, 0.068 mol MACl, and 0.034 mol TU in mixed solvent of DMF: NMP or DMF:DMSO. Blade coating of the perovskite precursor films was carried out on a commercial blade coater (ZAA2300.H from ZEHNTNER) using a ZUA 2000.100 blade (from ZEHNTNER) at room temperature in nitrogen‐filled glovebox. 20 µL precursor solution was used for blade deposition on each substrate. The gap for solution load between the substrate and blade was fixed at 200 µm. Once the precursor solution spread onto the substrate by blade coating, the liquid precursor film was transferred into a vacuum chamber, which was pumped to 1000 Pa in 15 s and stayed at the pressure for 2 min. Subsequently, the film was brought out of the vacuum chamber and annealed at 100 °C for 10 min in the glovebox to fully crystallize the film.

### Solar Cell Fabrication

The prepatterned glass/ITO and PEN/ITO substrates (OPV Tech Co., Ltd.) were sequentially cleaned by sonicating the substrates in acetone and isopropanol for 10 min each. The PEDOT:PSS hole transporting layer was spin coated from diluted PEDOT:PSS water solution at 4000 rpm for 30 s and the film was then annealed at 120 °C for 5 min in ambient air. After that, PTAA layer was spin coated from 5 mg mL^−1^ CB solution on PEN/ITO substrate at 5000 rpm for 30 s and annealed at 120 °C for 5 min in ambient air. The substrate was transferred to a nitrogen‐filled glovebox after it cooled down to room temperature. The perovskite absorber layer was subsequently deposited using the vacuum‐assisted blade‐coating method as described above. On top of perovskite film, the electron transporting layer PC_61_BM (20 mg mL^−1^ in chlorobenzene) and the interfacial layer BCP (2.5 mg mL^−1^ in isopropanol) were successively deposited by spin coating at 2000 rpm for 30 s and 5000 rpm for 30 s, respectively. Finally, 100 nm Ag contact was deposited by thermal evaporation. The active areas of the solar cells were 0.09 and 1 cm^2^, respectively, for the small‐size and large‐area devices, which were determined by the overlapping between the top Ag and bottom ITO electrode. All the devices for performance and stability evaluation were tested without encapsulation.

### Characterizations

Morphologies of the perovskite films were imaged with a scanning electron microscope (SEM, FEI Apreo LoVac). The crystal structure was characterized by Bruker D8 Advance X‐ray diffractometer (XRD) with Cu K*α* radiation at 40 kV and 40 mA. PL lifetime was measured by the time‐correlated single photon counting method with an Edinburgh Instruments FLS980 fluorescence spectrometer. The excitation source used was a picosecond pulsed diode laser at 532 nm.

The current density‒voltage (*J‒V*) curves of PSCs were recorded using a Keithley 2400 source measurement unit and a Newport solar simulator (ORIEL‐SOI3A) with an AM1.5G spectrum. All of flexible photovoltaic devices were measured in the flatten state. The light intensity was adjusted to 100 mW cm^−2^ using standard Si cell (91150V). Both forward and reverse scans were measured with the scanning speed of 0.15 V s^−1^. The EQE spectra were measured in DC mode on a spectrum corresponding system (Enlitech QE‐R), calibrated by Si reference solar cell.

The EL spectrum and ERE of the perovskite LED were recorded simultaneously by a commercialized system (XPQY‐EQE‐350‐1100, Guangzhou Xi Pu Optoelectronics Technology Co., Ltd.) that is equipped with an integrated sphere (GPS‐4P‐SL, Labsphere) and a photodetector array (S7031‐1006, Hamamatsu Photonics).

## Conflict of Interest

The authors declare no conflict of interest.

## Supporting information

Supporting Information

## Data Availability

Research data are not shared.
