# Supplementary material for: An Embedding 2D/3D Heterostructure Enables High‐Performance FA‐Alloyed Flexible Perovskite Solar Cells with Efficiency over 20%
Source: Adv Sci (Weinh). 2021 Oct 8;8(22):2101856. doi: 10.1002/advs.202101856 (PMC8596135; doi:10.1002/advs.202101856)
Supplement: Supplementary file 1 — Supporting Information [file ADVS-8-2101856-s001.pdf]

© 2021 Wiley-VCH GmbH

ADVANCED SCIENCE Open Access

Supporting Information

for *Adv. Sci.*, DOI: 10.1002/advs.202101856

An Embedding 2D/3D Heterostructure Enables Efficient and Stable FA-alloyed Flexible Perovskite Solar Cells

Zhen Wang[1], Yuanlin Lu[2], Zhenhua Xu[1], Jinlong Hu[1], Yijun Chen[1], Cuiling Zhang[1], Fei Guo[1,3] and Yaohua Mai[1]**

Supporting information

An Embedding 2D/3D Heterostructure Enables Efficient and Stable FA-alloyed Flexible Perovskite Solar Cells

Zhen Wang[1], Yuanlin Lu[2], Zhenhua Xu[1], Jinlong Hu[1], Yijun Chen[1], Cuiling Zhang[1], Fei Guo[1,3] and Yaohua Mai[1]**

[1]Institute of New Energy Technology, College of Information Science and Technology, Jinan University, Guangzhou, 510632, China

[2]Institute of Polymer Optoelectronic Materials & Devices State Key Laboratory of Luminescent Materials & Devices, South China University of Technology, Guangzhou, 510640, P. R. China

[3]Key Laboratory of Advanced Material Processing & Mold (Ministry of Education), Zhengzhou University, Zhengzhou, 450002, China

Correspondence: F. G. (email: fei.guo@jnu.edu.cn) and Y. M. (email: yaohuamai@jnu.edu.cn)

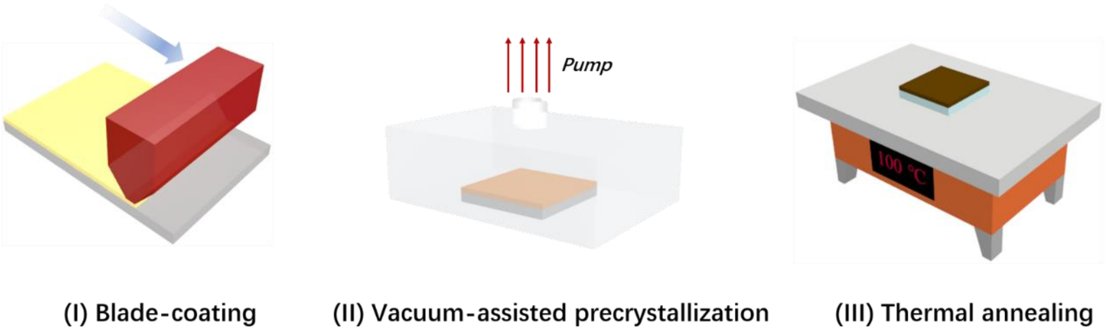

Figure S1. Schematic illustration of the one-step deposition of a perovskite film via blade coating.

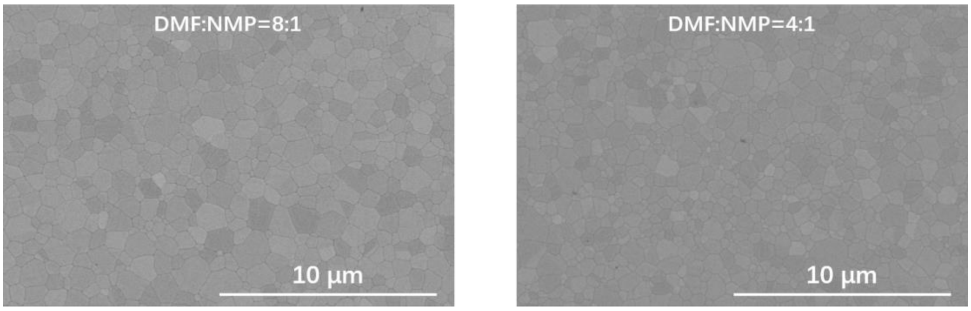

Figure S2. SEM images of perovskite films fabricated via mixed solution for DMF:NMP=8:1 and DMF:NMP=4:1 (volume ratio).

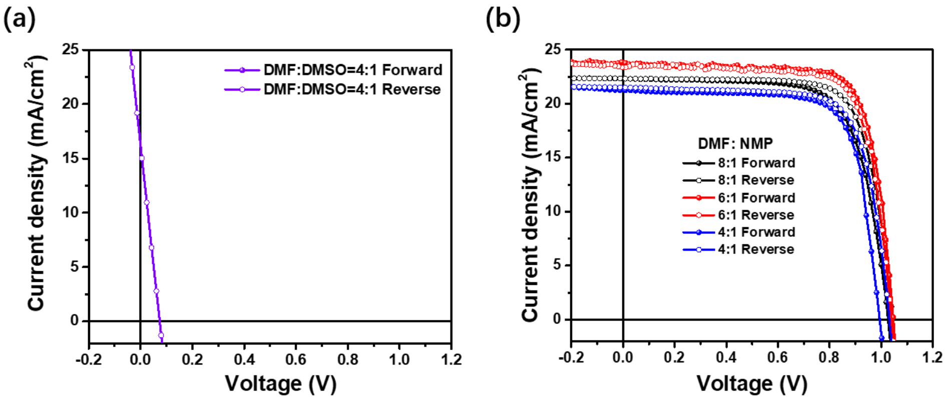

Figure S3. *J-V* curves of PSCs processed from the mixed solvent of (a) DMF:DMSO = 4:1 and (b) DMF:NMP of different volume ratios.

Table S1. Performance data for PSCs based on the perovskite precursor solution for DMF:DMSO = 4:1 (volume ratio).

DMF:DMSO		J_{SC} [mA/cm^2]	V_{OC} [V]	FF [%]	PCE [%]
4:1	Forward	14.40	0.08	25.92	0.30
	Reverse	14.25	0.08	25.87	0.29

Table S2. Performance data for PSCs based on different volume ratio of DMF:NMP perovskite precursor solution.

DMF:NMP		J_{SC} [mA/cm^2]	V_{OC} [V]	FF [%]	PCE [%]
8:1	Forward	22.32	1.02	74.39	16.94
	Reverse	22.41	1.04	76.23	17.77
6:1	Forward	23.67	1.04	75.13	18.49
	Reverse	23.61	1.03	73.56	17.93
4:1	Forward	21.23	1.01	74.08	15.88
	Reverse	21.51	1.02	74.92	16.43

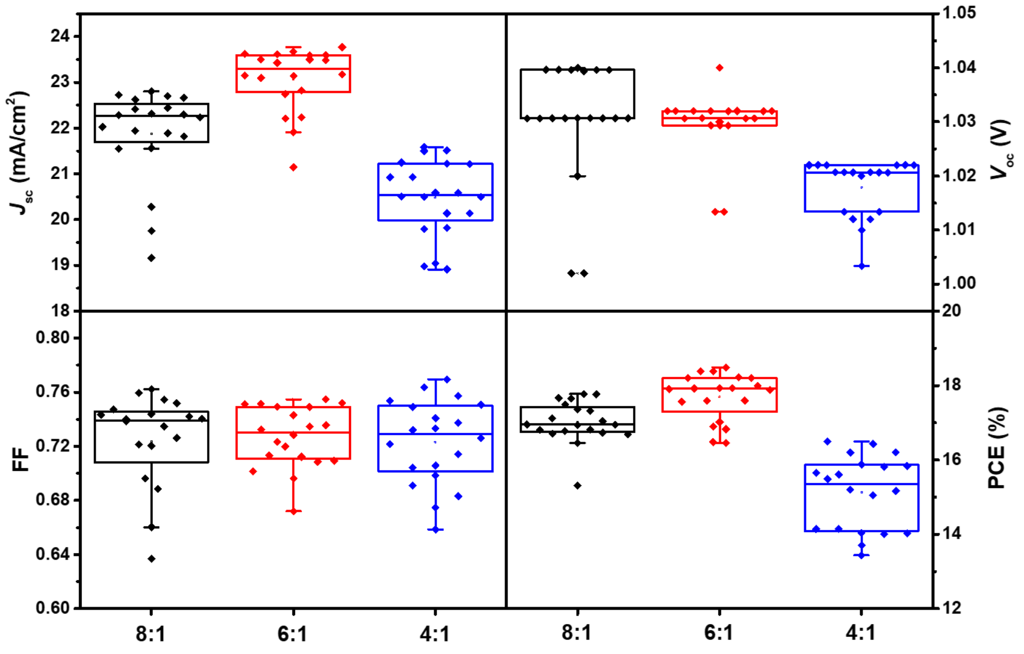

Figure S4. Statistic J_{SC}, V_{OC}, FF and PCE of the PSCs fabricated via mixed solution with different volume ratios of DMF:NMP.

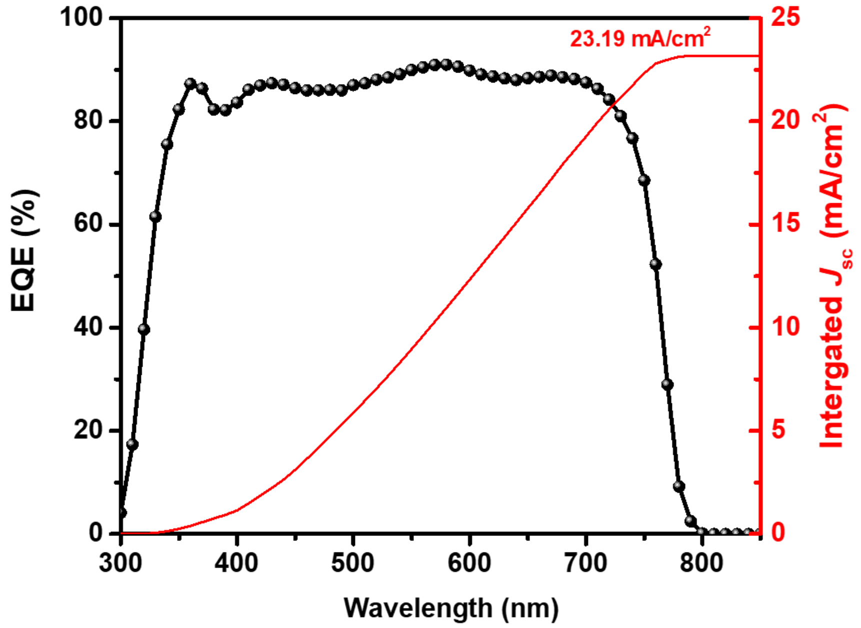

Figure S5. EQE spectra of the PSC fabricated by mixed solvent for DMF:NMP = 6:1 (volume ratio).

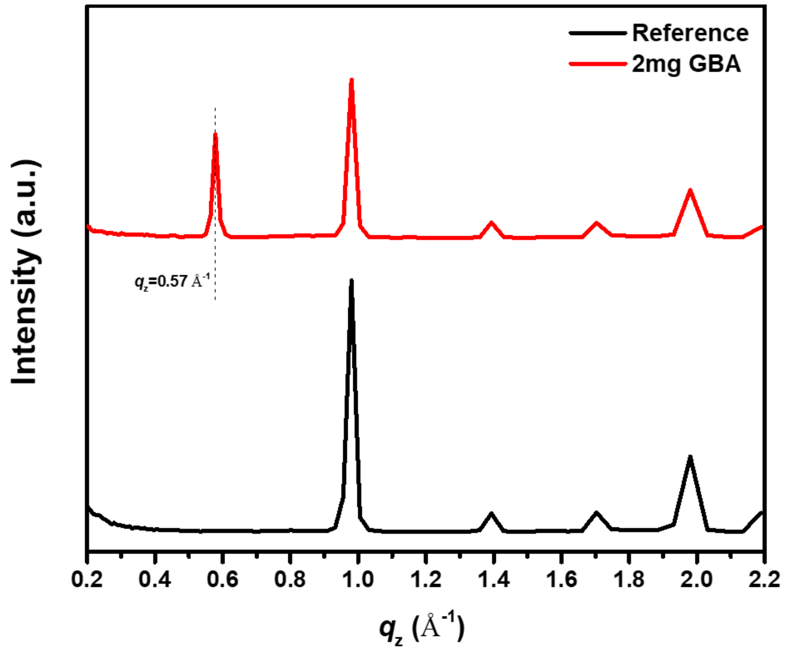

Figure S6. Out-of-plane line-cut profiles in GIWAX for perovskite films of reference and containing 2 mg/mL GBA.

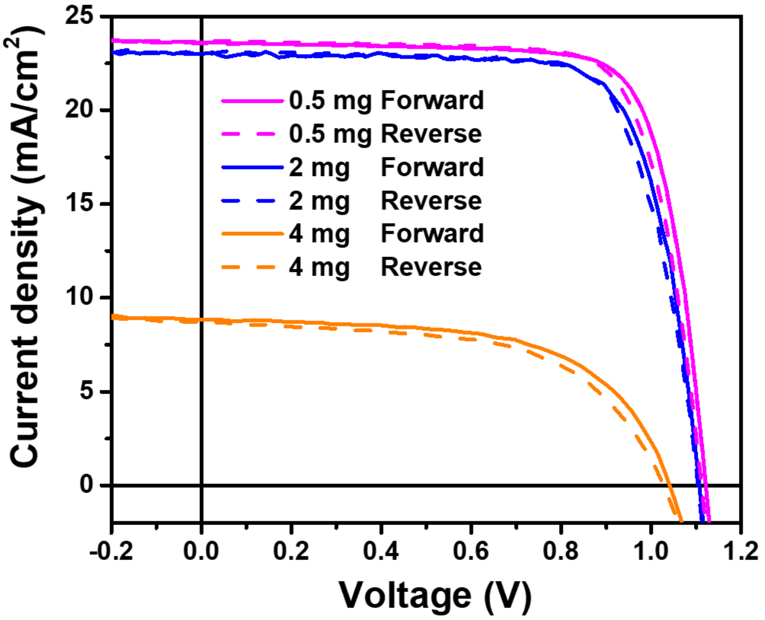

Figure S7. *J*–*V* curves for PSCs fabricated via perovskite precursor solution containing 0.5 mg/mL, 2 mg/mL and 4 mg/mL.

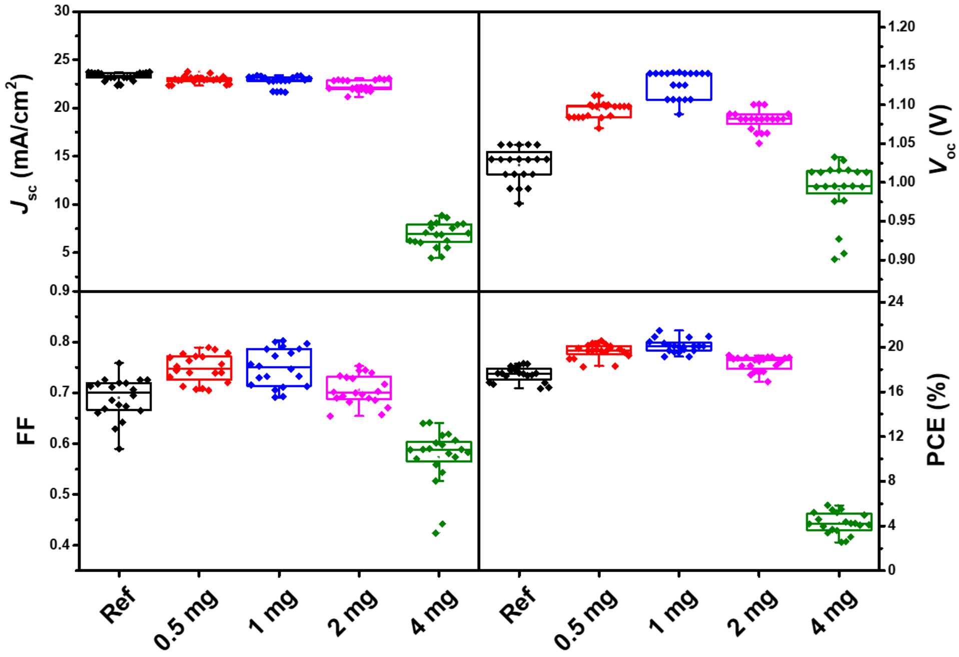

Figure S8. Statistic J_{SC}, V_{OC}, FF and PCE of the PSCs fabricated via perovskite precursor solution containing different concentration of GBA (mg/mL).

Table S3. Values for time-resolved PL characteristics by fitting decay curves of different samples.

Sample	A_1	τ_1 [ns]	A_2	τ_2 [ns]	τ
Ref	1.67	16.29	0.36	86.12	58.21
2D/3D	0.38	18.41	1.07	336.72	367.27

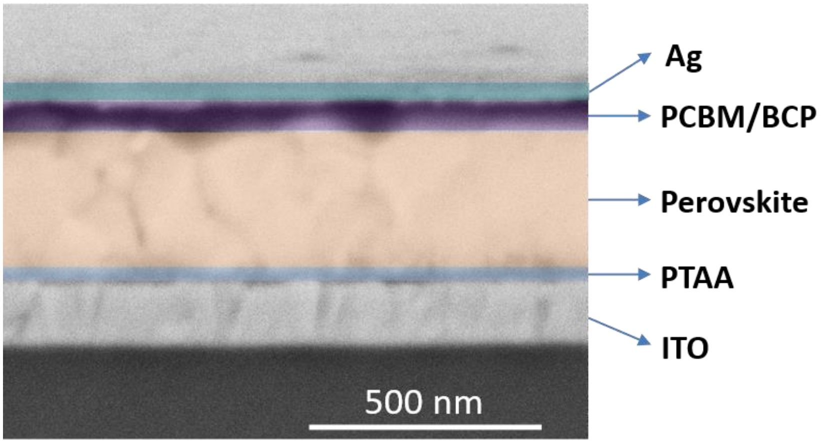

Figure S9. Cross-sectional SEM image of a PSC.

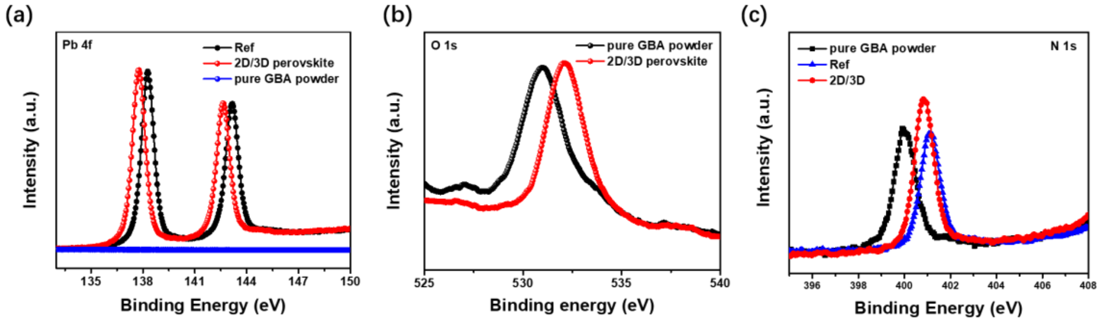

Figure S10. (a, b) The X-ray photoelectron spectroscopy (XPS) spectra of Pb and O signals in perovskite films, respectively. (c) High-resolution N 1s spectra of pure GBA powder, 3D reference perovskite film and 2D/3D heterostructure film.

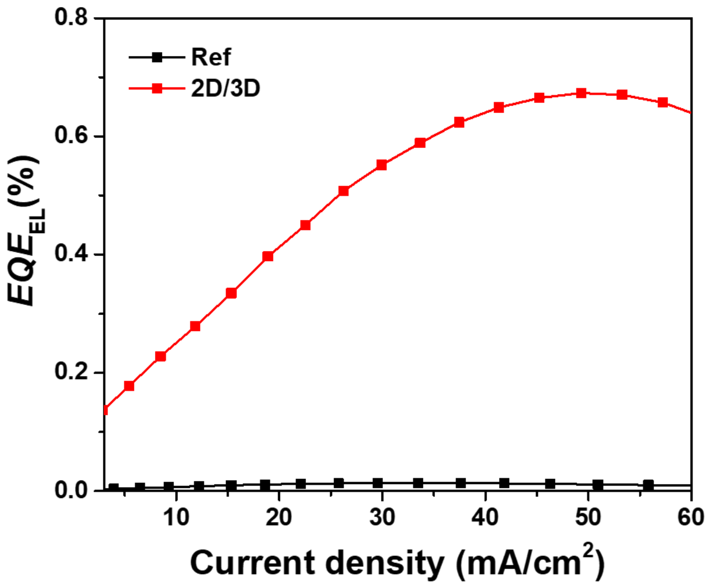

Figure S11. EQE_{EL} versus current density curves for reference and 2D/3D devices.

Table S4. Performance data for *f*-PSCs fabricated via precursor solution containing different concentration of GBA.

GBA concentration [mg/mL]		J_{SC} [mA/cm^2]	V_{OC} [V]	FF [%]	PCE_{Max} [%]	PCE_{Avg} [%]
Ref	Forward	22.83	1.04	75.08	17.83	16.66
	Reverse	22.58	1.04	72.61	17.05	
1	Forward	22.36	1.13	79.68	20.13	19.08
	Reverse	22.25	1.13	80.19	20.16	
2	Forward	21.61	1.09	72.63	17.11	16.42
	Reverse	21.59	1.09	74.73	17.57	

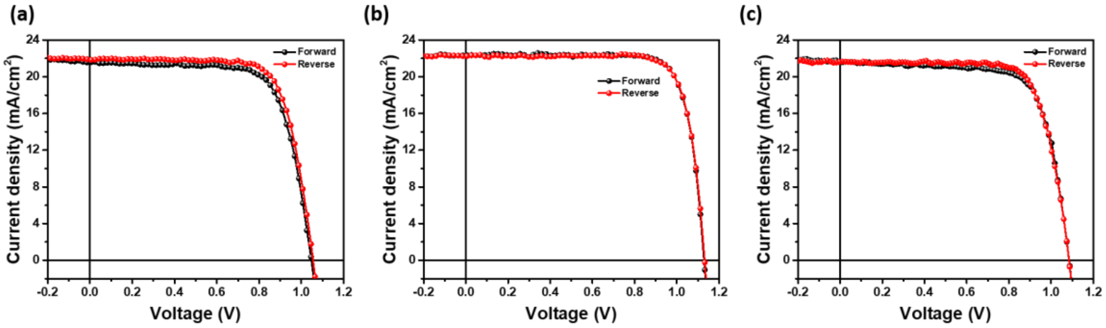

Figure S12. *J–V* curves for *f*-PSCs fabricated via perovskite precursor solution containing (a) 0 mg/mL (Ref), (b) 1 mg/mL, and (c) 2 mg/mL GBA.

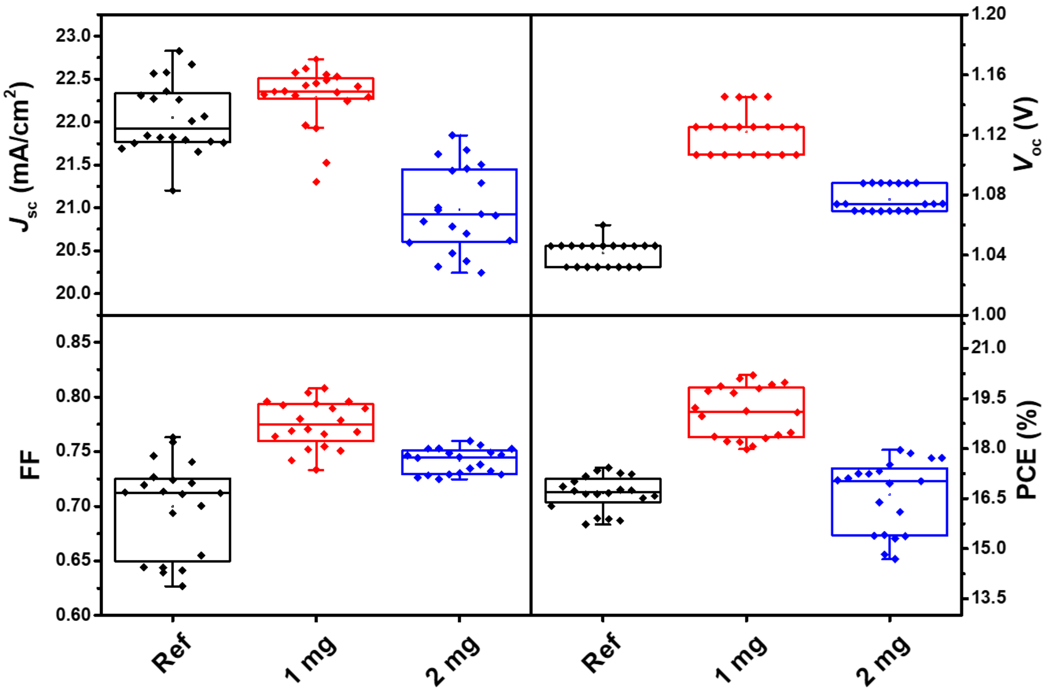

Figure S13. Statistic J_{SC}, V_{OC}, FF and PCE of the *f*-PSCs fabricated via perovskite precursor solution containing different concentration of GBA (mg/mL).

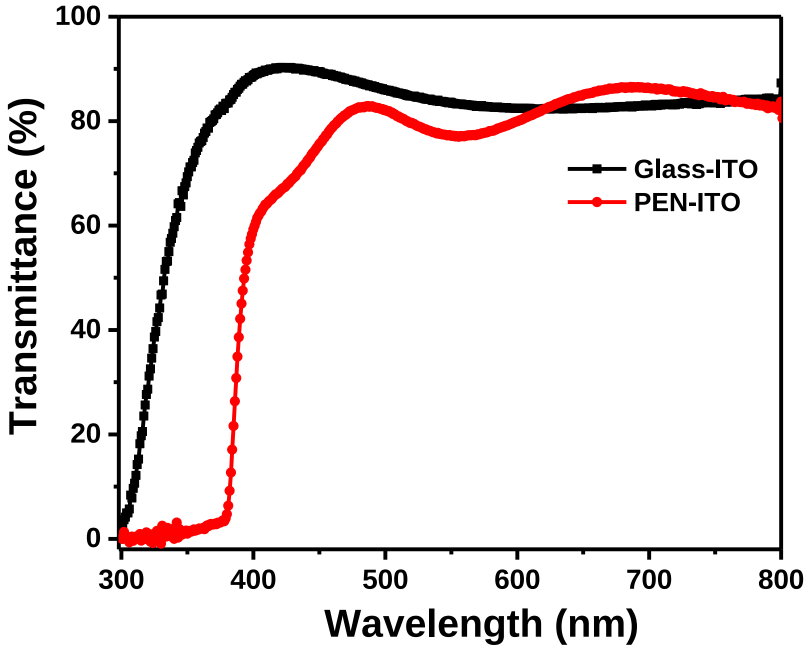

Figure S14. Transmission spectra of glass/ITO and PEN/ITO substrates.

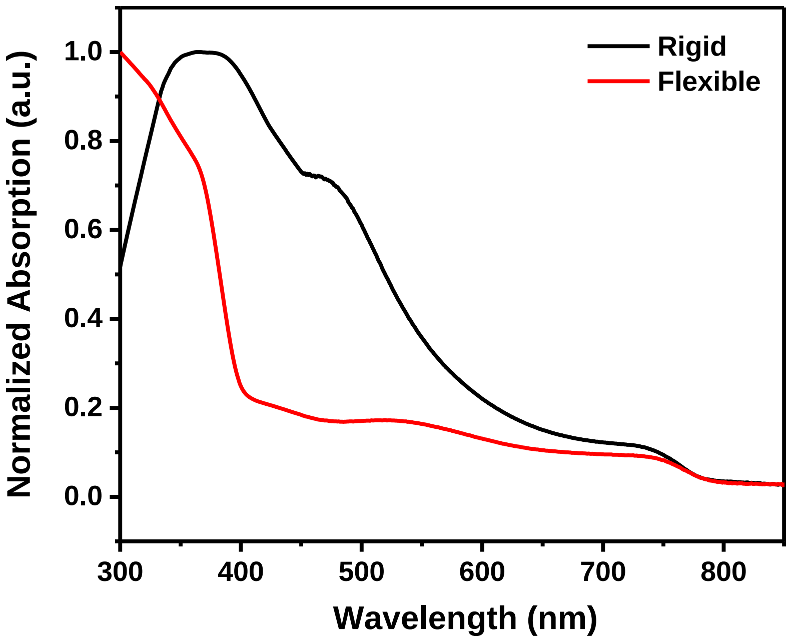

Figure S15. Absorption spectra of perovskite films deposited on the glass/ITO and PEN/ITO substrates.

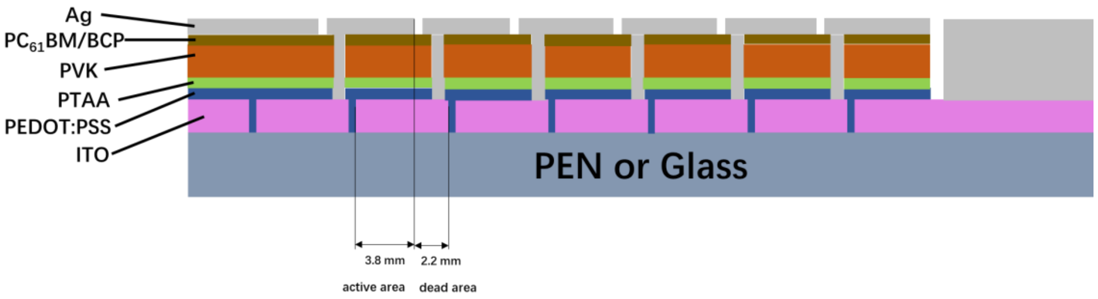

Figure S16. The schematic diagram of rigid or flexible perovskite solar module.

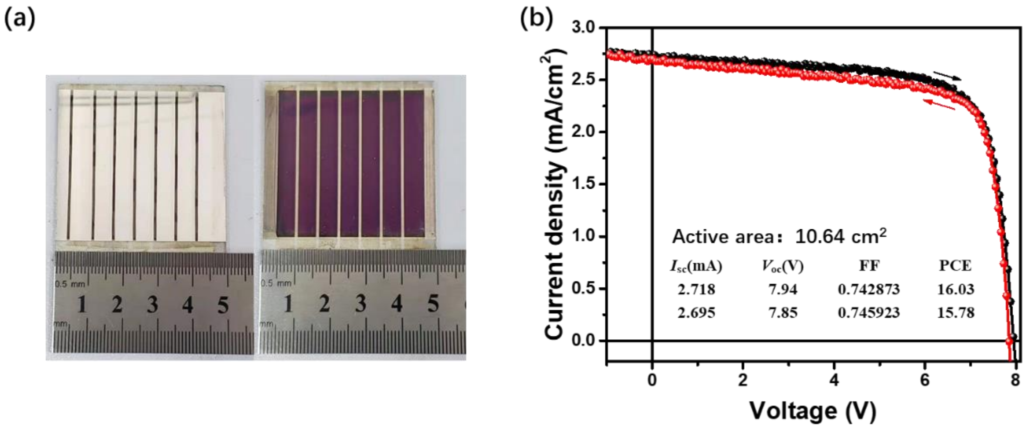

Figure S17. (a) Photograph and (b) *J*-*V* curve for rigid perovskite solar module with 5 ×5 cm.
